# Non-invasive diagnostic tool for Parkinson’s disease by sebum RNA profile with machine learning

**DOI:** 10.1038/s41598-021-98423-9

**Published:** 2021-09-20

**Authors:** Yuya Uehara, Shin-Ichi Ueno, Haruka Amano-Takeshige, Shuji Suzuki, Yoko Imamichi, Motoki Fujimaki, Noriyasu Ota, Takatoshi Murase, Takayoshi Inoue, Shinji Saiki, Nobutaka Hattori

**Affiliations:** 1 Biological Science Research, Kao Corporation, Tochigi, Japan; 2 Department of Dermatology and Allergology, Juntendo University Graduate School of Medicine, Tokyo, Japan; 3 Department of Neurology, Juntendo University School of Medicine, Tokyo, Japan; 4 Preferred Networks, Inc., Tokyo, Japan

**Keywords:** Genetics, Biomarkers, Neurology

## Abstract

Parkinson's disease (PD) is a progressive neurodegenerative disease presenting with motor and non-motor symptoms, including skin disorders (seborrheic dermatitis, bullous pemphigoid, and rosacea), skin pathological changes (decreased nerve endings and alpha-synuclein deposition), and metabolic changes of sebum. Recently, a transcriptome method using RNA in skin surface lipids (SSL-RNAs) which can be obtained non-invasively with an oil-blotting film was reported as a novel analytic method of sebum. Here we report transcriptome analyses using SSL-RNAs and the potential of these expression profiles with machine learning as diagnostic biomarkers for PD in double cohorts (PD [n = 15, 50], controls [n = 15, 50]). Differential expression analysis between the patients with PD and healthy controls identified more than 100 differentially expressed genes in the two cohorts. In each cohort, several genes related to oxidative phosphorylation were upregulated, and gene ontology analysis using differentially expressed genes revealed functional processes associated with PD. Furthermore, machine learning using the expression information obtained from the SSL-RNAs was able to efficiently discriminate patients with PD from healthy controls, with an area under the receiver operating characteristic curve of 0.806. This non-invasive gene expression profile of SSL-RNAs may contribute to early PD diagnosis based on the neurodegeneration background.

## Introduction

Parkinson's disease (PD) is a progressive neurodegenerative disease that is characterized by both motor symptoms and non-motor symptoms, such as depression, dysosmia, and dysautonomia (e.g., constipation and orthostatic hypotension)^1,2^. The prevalence of PD is 100–300/100,000 in any country^3^. The characteristic pathophysiology of PD includes depigmentation in the midbrain substantia nigra, neuronal loss, and α-synuclein-positive inclusion bodies—known as Lewy bodies—in the residual neuronal cytoplasm. Additionally, the deposition of α-synuclein in the autonomic nerves is a PD-specific finding, especially in the cardiac plexus, enteric plexus, and subcutaneous nerves^4–7^.

Facial seborrhea dermatitis, caused by excessive sebum secretion, is highly prevalent in PD and occasionally responds to levodopa therapy^8,9^. In addition to systemic neuroendocrine changes, including higher levels of androgen and prolactin, dysautonomia is partly associated with seborrhea^10,11^. Moreover, plasma/serum metabolic changes^12–14^ lipid composition, and fatty acid β-oxidation in sebum as well as volatile components of the skin are all PD-specific changes^15,16^. Sebum, a major component of skin surface lipids (SSLs), is released by a holocrine mode of secretion from sebaceous gland cells releasing their cellular components^17^. Measurable quantities of human coding and non-coding RNAs are found in SSLs (SSL-RNAs) because of RNase inhibition by the surrounding lipids^18^. SSL-RNAs can be non-invasively obtained using a single oil-blotting film, and AmpliSeq, a transcriptome method that uses target sequencing of over 20,000 human transcripts, can then be applied to detect SSL-RNAs. These techniques can be used to understand the physiological state of skin^18^. For example, SSL-RNA analysis of atopic dermatitis identified genes consistent with known pathophysiology and also indicated mechanisms related to altered lipid metabolism^18^. We therefore investigated whether the SSL-RNA transcriptome can non-invasively differentiate patients with PD from healthy controls using machine learning.

## Results

### Participant and transcriptome characteristics

**Figure 1:**
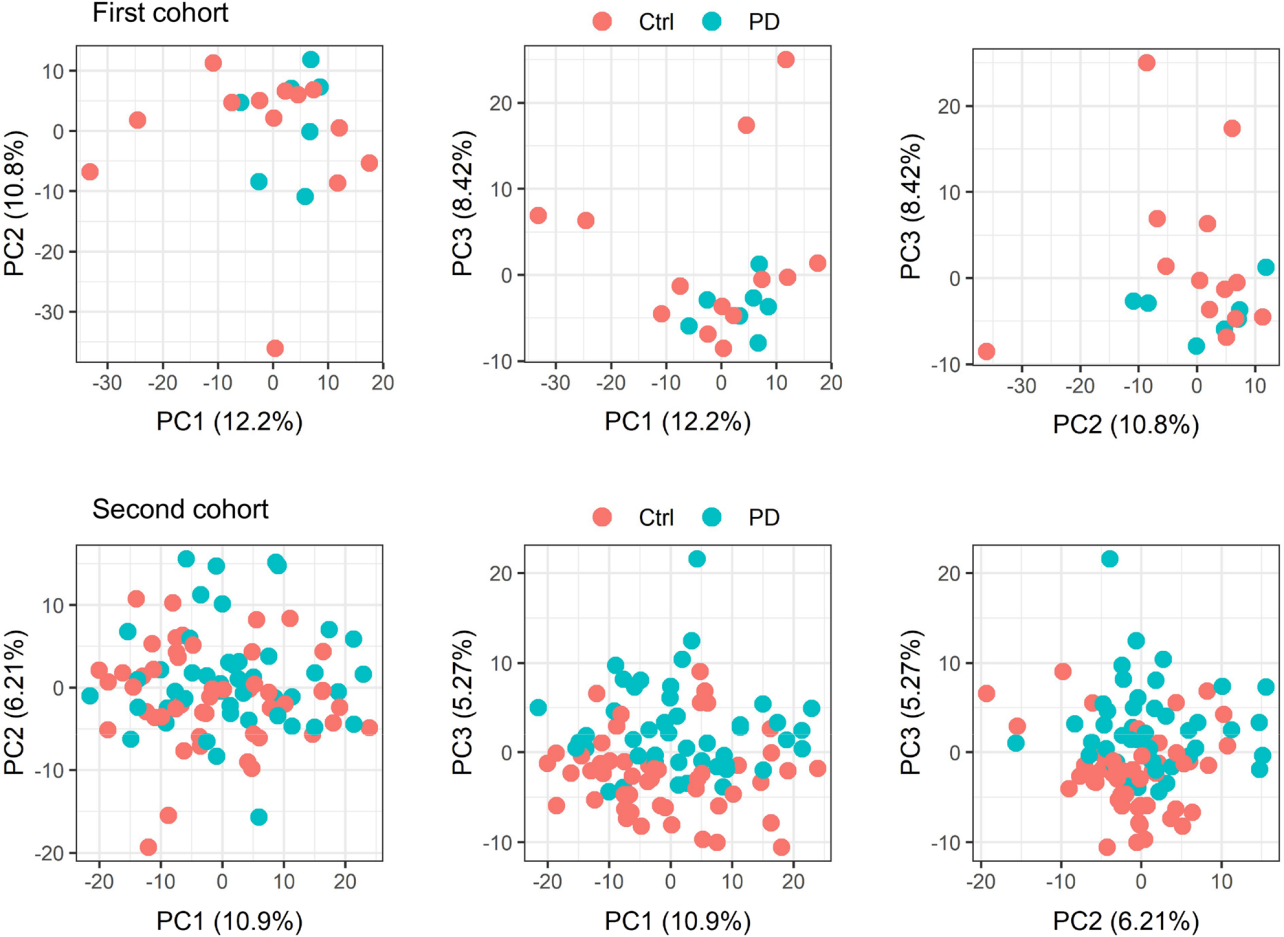
Principal component analysis using SSL-RNA profiles. The panels show two-dimensional principal component analysis plots with PC1–PC2, PC1–PC3, and PC2–PC3, respectively. *Ctrl* healthy controls, *PD* Parkinson’s disease, *PC* principal component. The plots were constructed using ggplot2 R package^19^.

To confirm whether SSL-RNAs include molecular changes associated with PD, we conducted two cohort studies: the first cohort included de novo female and male patients, and the second cohort included male patients who were taking antiparkinsonian medication or were drug naïve (Table 1). Because there were sample-dependent variations in the quality of SSL-RNAs in this study, the samples and genes for analysis were selected using several criteria (Supplementary Fig. S1, see also Data analysis section in “Methods”). As a result, 20 samples and 3768 genes were selected for analysis in the first cohort, and 96 samples and 4685 genes were selected for analysis in the second cohort. After selection, the first cohort consisted of 13 healthy controls and seven patients with PD, and the second cohort contained 50 healthy controls and 46 patients with PD. There were no significant differences in age between the healthy controls and patients with PD in either cohort (first cohort: *p* = 0.63, second cohort: *p* = 0.22, by unpaired Student’s t-test, Supplementary Table S1). Each cohort consisted of Hoehn and Yahr (H&Y) stage 2 or lower, with a mean H&Y stage of 1.0 in the first cohort and 1.5 in the second cohort. After normalization, the uniformity of SSL-RNA expression profiles in the selected samples and genes were confirmed by probability density distribution (Supplementary Fig. S2). Principal component analysis was performed to characterize the SSL-RNA profiles of patients with PD. In each cohort, we were unable to distinguish the patients with PD from the healthy controls using principal component (PC)1 and PC2; however, PC3 was able to distinguish between them (Fig. 1). Thus, the SSL-RNA expression profiles of PD had different characteristics from the profiles of healthy controls.

**Table 1:** Characteristics of participants.

	First cohort		Second cohort	
	Healthy control	PD	Healthy control	PD
Number	15	15	50	50
Female:male	9:6	9:6	0:50	0:50
Age (mean ± SD)	62.2 ± 11.8	61.7 ± 11.4	66.8 ± 9.08	64.2 ± 10.6
H&Y (cases)	(NA)	I (12), II (3)	(NA)	0 (2), I (20), II (28)
H&Y (mean ± SD)	(NA)	1.20 ± 0.400	(NA)	1.52 ± 0.574
Duration (year, mean ± SD)	(NA)	0.867 ± 0.562	(NA)	5.42 ± 4.27
De novo patients (cases)	(NA)	15	(NA)	8

*NA* not available, *SD* standard deviation.

### Differential expression analysis and gene ontology analysis

**Figure 2:**
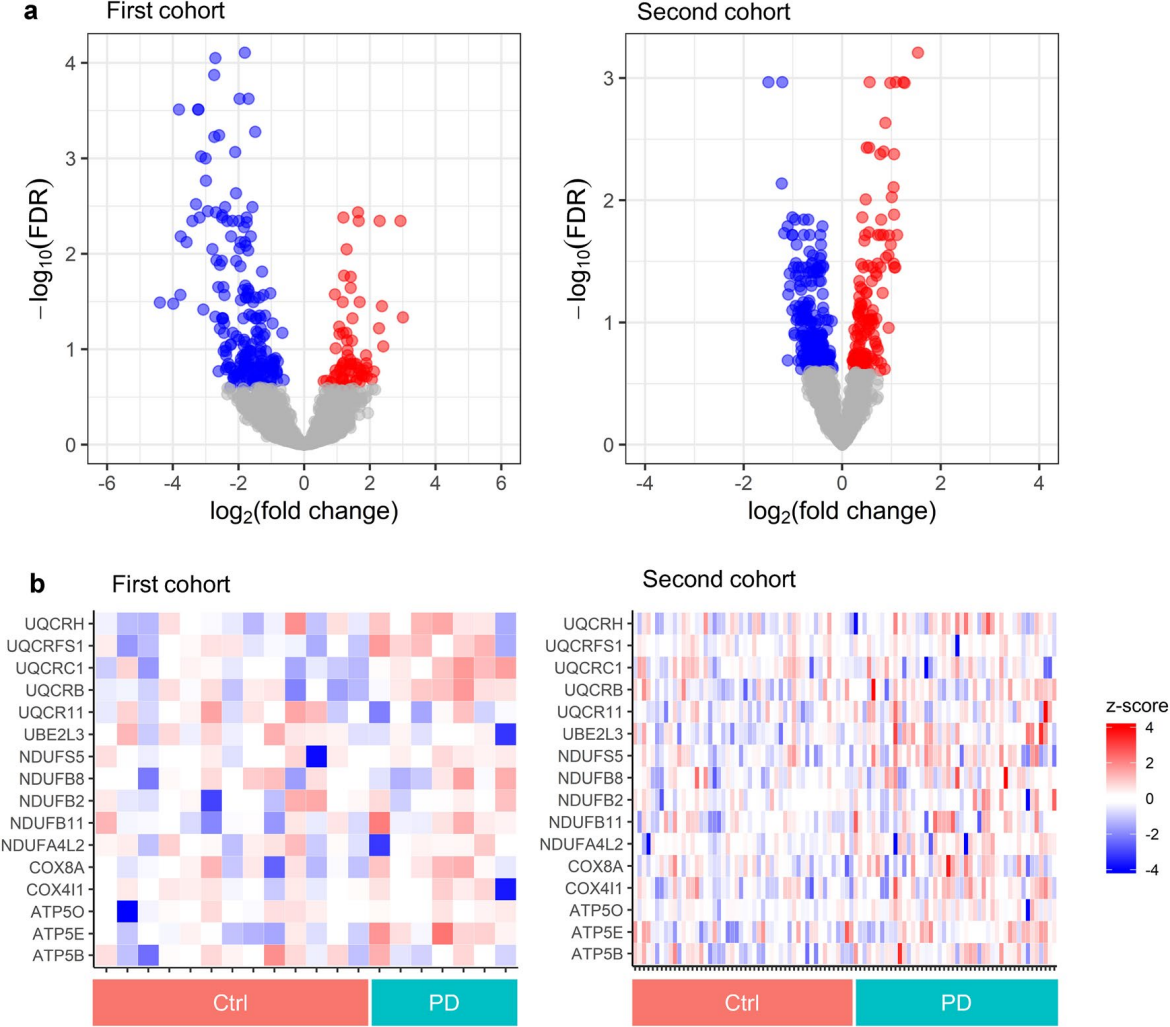
Differential expression analysis using SSL-RNAs in PD. (**a**) Volcano plot of expression changes in PD. In the first cohort, 209 genes were downregulated (blue) and 74 genes were upregulated (red). In the second cohort, 308 genes were downregulated (blue) and 151 genes were upregulated (red). Genes with FDR < 0.25 were considered to have a significant change. (**b**) Heatmaps of oxidative phosphorylation-related genes. Expression levels were converted to *z*-scores by genes. The gray color indicates missing values. *Ctrl* healthy controls, *FDR* false discovery rate, *NA* not available, *PD* Parkinson’s disease. The plots were constructed using ggplot2 and gplots R package^19,24^.

For insight into the biological characteristics of the SSL-RNA profiles in PD, we performed differential expression analysis between patients with PD and healthy controls. We set the false discovery rate (FDR) threshold at < 0.05, which is the de facto standard threshold for biological studies, and identified 95 differentially expressed genes (DEGs) in the first cohort and 91 DEGs in the second cohort. Of these, 80 were downregulated and 15 were upregulated in the first cohort, and 49 were downregulated and 42 were upregulated in the second cohort. Biological process and Kyoto Encyclopedia of Genes and Genomes (KEGG)^20,21^ pathway analyses using the Database for Annotation, Visualization and Integrated Discovery (DAVID)^22,23^ did not yield a common Gene Ontology (GO) term or KEGG pathway for both cohorts (Supplementary Table S2). Because the first and second cohorts had different study designs, we extracted the DEGs with an FDR threshold of < 0.25 to analyze the PD-related phenomena using SSL-RNAs in a more exploratory manner. Although the use of this threshold results in a five-fold increase in the false-positive rate of DEGs within each cohort, it allowed us to examine a wide range of changes that were common to both cohorts. A likelihood ratio test using DESeq2 yielded 283 and 459 DEGs in the first and second cohorts, respectively, of which 209 were downregulated and 74 were upregulated in the first cohort, and 308 were downregulated and 151 were upregulated in the second cohort (Fig. 2a). Although all participants in the second cohort were male, seven controls and one PD patient were female in the first cohort. To exclude sex difference effects, we reanalyzed the male participants of the first cohort and compared the results with those of all participants. Seventy-seven percent of DEGs were common between the two analyses, which was statistically significant (p < 0.01 by Fisher’s exact test) and indicates little impact on gene expression (Supplementary Fig. S3). In the GO analysis using DAVID were ‘Ribosome’ (hsa03010), ‘Endocytosis’ (hsa04144), ‘Viral carcinogenesis’ (hsa05203), ‘Renal cell carcinoma’ (hsa05211), and ‘Herpes simplex infection’ (hsa05168) in the first cohort’s downregulated genes. In the first cohort’s upregulated genes, the top five pathways were ‘Non-alcoholic fatty liver disease’ (hsa04932), ‘Huntington's disease’ (hsa05016), ‘Oxidative phosphorylation’ (hsa00190), ‘Parkinson’s disease’ (hsa05012), and ‘Alzheimer's disease’ (hsa05010). In the second cohort’s downregulated genes, the top five pathways were ‘Chemokine signaling pathway’ (hsa04062), ‘Neurotrophin signaling pathway’ (hsa04722), ‘Osteoclast differentiation’ (hsa04380), ‘Lysosome’ (hsa04142), and ‘Viral carcinogenesis’ (hsa05203). In the second cohort’s upregulated genes, the top five pathways were ‘Ribosome’ (hsa03010), ‘Oxidative phosphorylation’ (hsa00190), ‘Parkinson's disease’ (hsa05012), ‘Alzheimer's disease’ (hsa05010), and ‘Huntington's disease’ (hsa05016) (Supplementary Table S3). Notably, the two cohorts shared common KEGG pathways for neurodegenerative diseases, including PD, Huntington's disease, and Alzheimer's disease. The genes included in the KEGG pathway of ‘Parkinson's disease’ (hsa05012) were *ATP5E*, *UQCRB*, *UQCRC1*, and *UQCRFS1* in the first cohort, and *ATP5B*, *ATP5O*, *COX4I1*, *COX8A*, *NDUFA4L2*, *NDUFB11*, *NDUFB2*, *NDUFB8*, *NDUFS5*, *UBE2L3*, *UQCR11*, and *UQCRH* in the second cohort. These genes are responsible for oxidative phosphorylation in mitochondria and did not show consistent changes in each sample, but there was a modest tendency for their expression to be higher in patients with PD than in healthy controls (Fig. 2b). In the GO analysis for biological process using DAVID, we also identified processes related to mitochondrial function, such as ‘mitochondrial electron transport, ubiquinol to cytochrome c’ (GO:0006122), and ‘oxidative phosphorylation’ (GO:0006119) in the first cohort, and ‘mitochondrial electron transport, NADH to ubiquinone’ (GO:0006120), ‘mitochondrial ATP synthesis coupled proton transport’ (GO:0042776), ‘mitochondrial respiratory chain complex I assembly’ (GO:0032981), and ‘ATP biosynthetic process’ (GO:0006754) in the second cohort (Supplementary Table S4). Taken together, these findings suggest that expression changes of SSL-RNAs in patients with PD reflect molecular information about mitochondrial function, which is closely related to PD pathogenesis.

### DEGs common to the two cohorts

**Figure 3:**
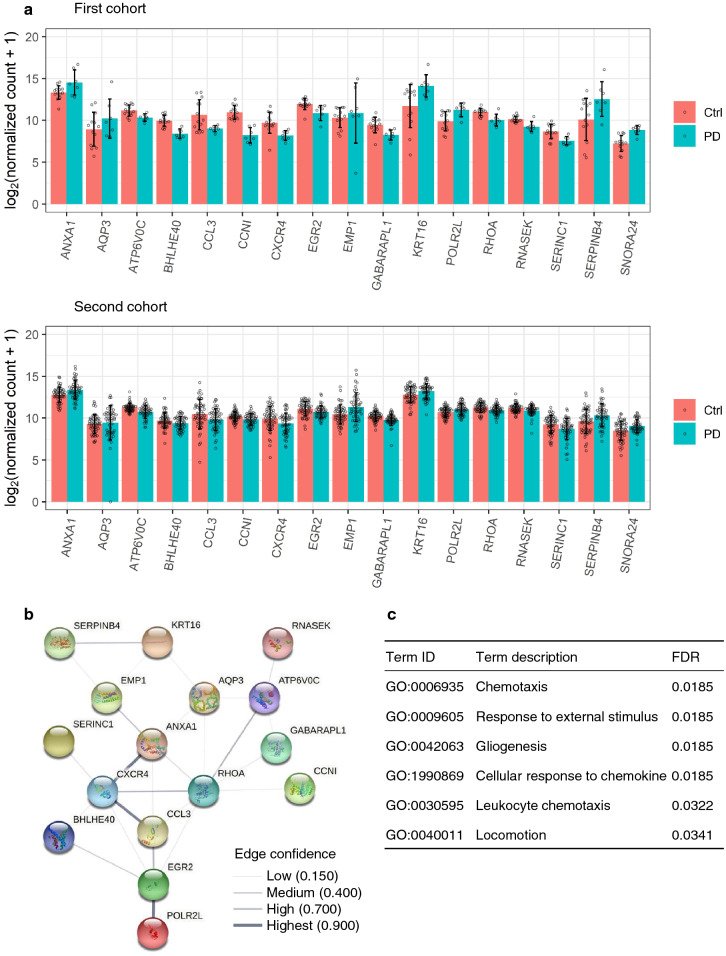
Reproducible genes and protein–protein interactions in both cohorts. (**a**) SSL-RNA expression levels of reproducible genes. Bar plots and error bars show the mean ± standard deviation (SD). Beeswarm plots represent the gene expression levels in each subject. (**b**) Protein–protein interaction analysis in 17 genes. STRING was used to depict the network. The thickness of the line represents the confidence of the edge. (**c**) Significant GO terms for biological process in 17 genes, yielded by STRING. *Ctrl* healthy controls, *FDR* false discovery rate, *PD* Parkinson’s disease. The plots were constructed using ggplot2 and ggbeeswarm R package^19,27^.

We next changed from analyzing trends in overall gene expression to focusing on several genes. Of 22 genes (*ATP13A2*, *ATXN3*, *CHCHD2*, *DNAJC13*, *FBXO7*, *GAK*, *GBA*, *GCH1*, *LRRK2*, *MAPT*, *PARK7*, *PINK1*, *POLG*, *PRKN*, *RAB29*, *RAB39B*, *SCARB2*, *SNCA*, *SREBF1*, *STX1B*, *SYNJ1*, and *VPS35*)^25^ involved in mutations and pathogenesis in familial and idiopathic PD, the expression of *CHCHD2* had a 2.28-fold increase in patients with PD (*p* = 3.02 × 10^−5^, FDR = 0.00417) in the first cohort (Supplementary Fig. S4). The DEGs that overlapped between the two cohorts were not statistically significant (Supplementary Fig. S5), but among the DEGs in our analysis, *ANXA1*, *AQP3*, *ATP6V0C*, *BHLHE40*, *CCL3*, *CCNI*, *CXCR4*, *EGR2*, *EMP1*, *GABARAPL1*, *KRT16*, *POLR2L*, *RHOA*, *RNASEK*, *SERINC1*, *SERPINB4*, and *SNORA24* were identified as common genes in both cohorts (Fig. 3a). In the GO analysis of biological process for these 17 genes using DAVID, the top five terms were ‘inflammatory response’ (GO:0006954), ‘granulocyte chemotaxis’ (GO:0071621), ‘monocyte chemotaxis’ (GO:0002548), ‘actin cytoskeleton reorganization’ (GO:0031532), and ‘calcium-mediated signaling’ (GO:0019722) (Supplementary Table S5). Using STRING^26^, a database that estimates protein–protein interactions, 16 of these genes (all except for *SNORA24*, which does not encode a protein) encoded proteins that were connected by edges and had relationships with each other. Eight of these genes (*CXCR4*, *CCL3*, *EGR2*, *ATP6V0C*, *RHOA*, *POLR2L*, *EMP1*, and *ANXA1*) were discontinuously connected with edges of moderate or greater confidence (Fig. 3b). The biological process analysis in STRING yielded ‘chemotaxis’ (GO:0006935), ‘response to external stimulus’ (GO:0009605), ‘gliogenesis’ (GO:0042063), ‘cellular response to chemokine’ (GO:1990869), ‘leukocyte chemotaxis’ (GO:0030595), and ‘locomotion’ (GO:0040011) (Fig. 3c). These results indicate that, with regard to SSL-RNA expression levels in patients with PD, changes in the immune system are a robust phenomenon that is common to both cohorts.

### Discrimination between patients with PD and healthy controls using machine learning

**Figure 4:**
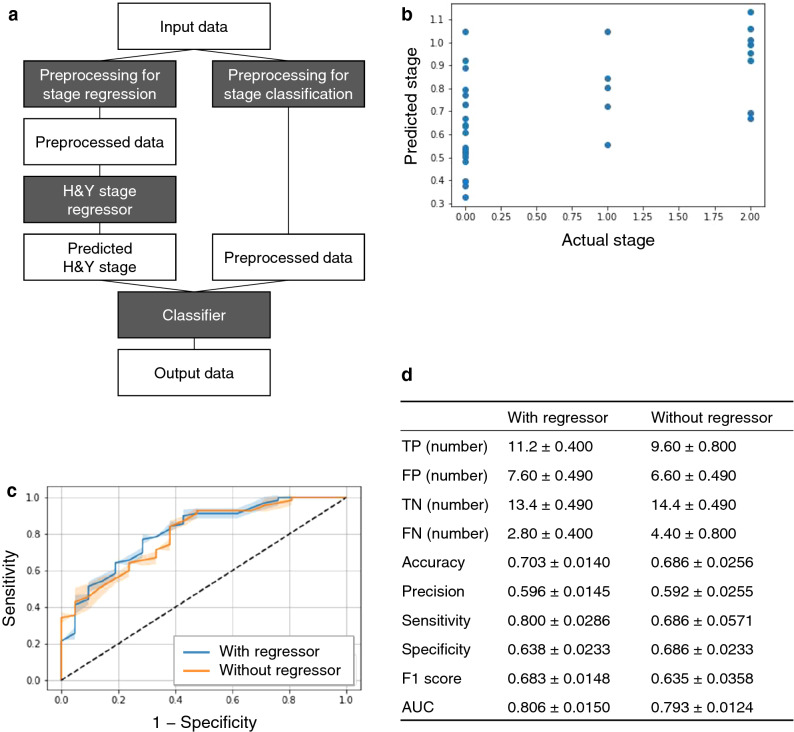
Discrimination model for Parkinson’s disease using machine learning with SSL-RNA profiles. (**a**) Discrimination pipeline. The rank normalized expression level, age, and sex were used as the input data. (**b**) Predicted H&Y stage with the regressor. Dots were plotted for each test sample showing the actual stage (*x*-axis) vs. the predicted stage (*y*-axis). (**c**) Receiver operating characteristic curve of the discrimination model. The graph shows 1 − specificity (*x*-axis) vs. sensitivity (*y*-axis) in the test samples. Lines and error bands represent the means and SDs of five receiver operating characteristic curves with different seeds. (**d**) Performance of the discrimination model. The performance was evaluated in the test samples. Each metric is shown as the mean and SD of the evaluation results of five trainings with different seeds. *AUC* area under the receiver operating characteristic curve, *H&Y* Hoehn and Yahr, *FN* false negative, *FP* false positive, *TN* true negative, *TP* true positive.

We examined the potential ability to discriminate between PD and healthy controls using machine learning with SSL-RNA profiles. Considering the small sample size of the first cohort, we combined both cohorts for machine learning analysis. The variables for machine learning are RNA expression levels, age at sampling, and gender because some gene expression may be affected by aging, and the male to female ratios are not homogenous in the two cohorts. In addition, to examine the effect of levodopa medication in the second cohort, we performed correlation analysis. We concluded that the levodopa medication effect was minor because only one gene (*FADS2*) correlated with levodopa dose. (Supplementary Table S6). After selecting parameters in the training samples, the performance of the discrimination model was evaluated in the test samples. The discrimination model was developed using extremely randomized trees (ERTs)^28^ and consisted of two models: the H&Y stage regressor and the classifier (Fig. 4a). For the H&Y stage regressor, the predicted stage correlated with the actual stage (Fig. 4b); the Spearman’s correlation coefficient was 0.604 (*p* = 1.24 × 10^–4^). We then used the predicted stage in the H&Y stage regressor and used the rank normalized expression level, age, and sex as inputs of classifier, and developed the discrimination model. Its performance was evaluated using the area under the receiver operating characteristic curve (AUC). This model was able to distinguish between patients with PD and healthy controls (AUC = 0.806); the performance of this model was better than that of the model without the H&Y stage regressor (AUC = 0.793) (Fig. 4c). In the discrimination model with the regressor, the sensitivity was considerably improved compared with that without the regressor (Fig. 4d). The F1 score, which is the harmonic mean of the sensitivity and precision, was also better in the model with the regressor. Together, these findings demonstrate that machine learning with SSL-RNA profiles can discriminate patients with PD from healthy controls. Moreover, the performance of the discrimination model can be improved using a combination of H&Y stage regressor and classifier.

## Discussion

In this double-cohort study, a principal component analysis with transcriptomic analysis using SSL-RNAs that were obtained non-invasively with a single oil-blotting film was able to identify the expression profiles of patients with PD compared with healthy controls. In each cohort, KEGG pathway analysis yielded changes in pathways for neurodegenerative diseases, including PD, and in those involved in mitochondrial function, which is closely associated with PD. The 17 common DEGs in both cohorts encoded a group of proteins that functionally and/or hypothetically interacted with one another and were associated with the immune system. Furthermore, ERT using SSL-RNA profiles as inputs allowed us to efficiently discriminate patients with PD from healthy controls, implying the potential use of SSL-RNAs in a novel, non-invasive method for the detection of PD.

PD patients exhibit elevated prolactin levels and excessive sebum production^8,10,29–35^. Both seborrhea and elevated prolactin levels in PD can be treated by restoring normal levels of dopamine (e.g., by levodopa or dopaminergic agonist administration)^9,31–33,35^. Moreover, both the expression of acetylcholine receptor α7 in the human sebaceous gland and cholinesterase activity around sebaceous gland tissue imply that the sebaceous glands may be regulated by cutaneous autonomic nerves^36,37^. Similarly, in PD especially, decreased somatosensory/autonomic nerve terminals and increased phosphorylated-α-synuclein (Ser129)-positive deposits have been identified in the sebaceous glands and neighboring structures^4,5,38–41^. Taken together, the profile changes of SSL-RNAs are likely to be PD-specific because of abnormalities in the neuroendocrine (prolactin–dopamine axis) and/or peripheral autonomic nervous systems. This suggests that their use as a potential biomarker might produce another possible use, as a response-associated biomarker to PD medication.

In each cohort of the present study, GO analysis of upregulated DEGs in patients with PD identified mitochondria-associated biological processes, containing genes encoding multiple subunits of the NADH:ubiquinone oxidoreductase supernumerary subunits (NDUF) complex, which is a component of complex I. Considering that deficiencies of complex I and II have been detected in nigral neurons in PD^42^, the increased expression of RNAs related to oxidative phosphorylation in SSL-RNAs might be derived from the upregulated energy metabolism of sebaceous cell mitochondria in response to neurotransmission changes and/or as compensation for mitochondrial dysfunction in sebum cells.

KEGG pathway analysis using the upregulated genes identified six pathways in both cohorts (oxidative phosphorylation, non-alcoholic fatty liver disease, Huntington's disease, Alzheimer's disease, PD, and cardiac muscle contraction). These pathways were extracted using a group of genes associated with oxidative phosphorylation in mitochondria. The patients analyzed in this study did not include patients with non-alcoholic fatty liver disease or dyslipidemia, but did include patients with diabetes (n = 4) and hypertension (n = 6). Thus, the confounding factor of liver disease was unable to be completely excluded. In addition, for a more extensive clinical indication, comparative analyses of PD with other parkinsonian disorders, such as progressive supranuclear palsy and multiple system atrophy, should be performed. Seventeen genes that were commonly altered in the two cohorts were associated with the immune system. Seborrheic skin, bullous pemphigoid, and rosacea have a high prevalence in PD and may produce an inflammatory state of the skin that is reflected in SSL-RNAs. Although patients with distinct eczema or inflammation were excluded from this study, the effects of skin diseases, inflammation in autonomic nerve terminals, and other systemic inflammatory conditions on SSL-RNAs need to be closely examined in the future. However, the expression changes in these 17 immune-related genes were confirmed in the two cohorts despite the different trial batches and treatment histories, and the functional relationship between the proteins in the STRING database analysis demonstrated that these changes in SSL-RNA expression levels are robust in PD. However, 16 out of 17 immune-related genes were not of high variable importance rank in the diagnostic model with the machine learning (data not shown). Whilst 17 common DEGs were identified, no genes with significant overlap were detected between cohorts. This might be because of various factors, such as the period of sample collection influencing sebum contents or anti-parkinsonian medication.

The performance of our proposed model for discriminating between PD and healthy controls was AUC = 0.806, which is comparable to that of blood and sebum metabolite analyses using expensive LC–MS techniques^12–16^. The SSL-RNA analysis has the technical advantage of being able to easily change the measurement of the selected genes from transcriptome to polymerase chain reaction. The metabolites and/or transcripts may allow for the early diagnosis of PD, and further improvements in accuracy can be expected when the two are combined. Furthermore, a non-invasive or minimally invasive multi-omics approach using the metabolome and transcriptome may enable the understanding of processes from gene expression to the consequences of metabolism, with continuous monitoring.

In the discrimination model for PD using machine learning with SSL-RNA profiles, the model with the regressor showed better performance than that without the regressor in the AUC evaluation. From this result, the prediction of H&Y stage is effective for discriminating between patients with PD and healthy controls. We thus speculate that it may become possible to distinguish between a subject with a high degree of certainty of PD and healthy controls using the prediction of H&Y stage regressor as the input. Taking these insights into consideration, the regressor for other scales, such as the Unified PD Rating Scale, might also be useful for the discrimination model. However, the sensitivity of the model with the regressor was improved in this study, but the specificity was not. We were unable to identify the precise reasons for this, and further analyses, such as the analysis of medical histories of the false-positive participants, may be needed. Other variables, such as on age at sampling, gender, and medication, should be examined in future studies.

Several limitations must be considered in this analysis of PD using SSL-RNAs. First, the study was conducted at a single facility for both patients with PD and healthy controls. In addition, 10% of the sample was excluded from the analysis at data cleaning, and a threshold of FDR < 0.25 was used as a lenient criterion for DEG analysis. Furthermore, the amount of SSL-RNAs that can be extracted from a single oil-blotting film is extremely low, and SSLs are located on the outermost layer of the body; thus, this technique will always be subject to influences from both inside and outside the body, and confounding factors can easily occur. Another limitation of this study is the small sample size of both cohorts. The results may be influenced by medication, disease severity, gender, and other factors. Thus, further investigation with larger cohorts should be performed. Although it is difficult to obtain overall reproducibility in in vivo omics analyses without rigorous study design and methodologies that consider batch effects and confounding factors^43^, this study aimed to explore the potential of SSL-RNA analysis in PD in small to medium cohorts. Therefore, in future it will be necessary to control the subjects' daily life and physical conditions, season, and time of collection in multiple facilities, and to conduct the study with a large enough sample size for a more accurate analysis. Additionally, SSLs mainly contain RNAs from the sebaceous glands, but also contain some RNAs from the epidermis and hair follicles, which makes it difficult to analyze the mechanisms identified in SSL-RNAs in vitro or using model organisms. In this context, validation studies to expand the variety of related diseases and disease stages are needed for future validation in humans and in vivo models, including dermal organoids.

In conclusion, our study proposed the new procedure for diagnosis of PD using the non-invasive SSL-RNA analysis in combination with machine learning. We further investigate the utility of this method in understanding of early pathogenesis of PD and discrimination ability among PD and other neurodegenerative diseases.

## Methods

### Ethics statement

This study protocol complied with the Declaration of Helsinki and was approved by the Ethics Committee of Juntendo University (#2019227) and the Human Research Ethics Committee of Kao Corporation (T190-190120). Written informed consent was obtained from all patients.

### Participants

Patients with PD were recruited at Juntendo University Hospital (Tokyo, Japan). Healthy controls were recruited at Inforward, Inc. (Tokyo, Japan). The first cohort was recruited from July 2018 to March 2019 for female and male de novo patients, and the second cohort was recruited from March 2019 to October 2019 for male patients with or without medication. All PD patients were diagnosed with clinically established PD by board-certified neurologists according to the 2015 Movement Disorders Society diagnostic criteria for PD^2^. Neither patients nor healthy controls had a current history of tumors, cancer, collagen vascular diseases, or skin diseases such as atopic dermatitis, acne vulgaris, or psoriasis. Participants using oral/topical antibiotics or with distinct facial eczema at the time of sampling were excluded (Table 1). Disease duration refers to the time since the initial motor symptoms of PD. H&Y stages were defined during the “on” phase for practical and ethical reasons.

### SSL collection and SSL-RNA preparation

The SSL collection and SSL-RNA purification were performed as described previously^18^. In addition to the RNA purification method described previously, we also used the following modified method for RNA preparation during the study. There were no differences in the transcriptome analysis results between the two methods. Briefly, the oil-blotting film was cut into small pieces followed by homogenization with 1.45 mL QIAzol (QIAGEN, Hilden, Germany). The supernatant (1.3 mL) was collected in a tube and 260 μL chloroform was added, and the mixture was vortexed for 10 s. After centrifuging at 12,000×*g* for 15 min at 4 °C, 750 μL of the upper phase was transferred to a fresh tube, and 750 μL 85% ethanol was added. RNA was purified according to the manufacturer’s instructions using the RNeasy Mini Kit and QIAcube (QIAGEN) with concomitant DNase I treatment. RNA was eluted in 100 μL nuclease-free water, concentrated by standard ethanol precipitation, and dissolved in 10 μL nuclease-free water.

### AmpliSeq transcriptome analysis

The transcriptome analysis using SSL-RNAs was performed as described previously^18^. Briefly, 1.5 µL RNA solution and an Ion AmpliSeq Transcriptome Human Gene Expression Kit (Thermo Fisher Scientific, Waltham, MA, USA) were used for the sequencing library preparation, with modified protocols. In addition to the aforementioned methods, we added 250 ng and 100 ng T4 Gene 32 Protein (New England Biolabs, Ipswich, MA, USA) to the reverse transcription reaction mix and the first round polymerase chain reaction mix, respectively. The templates for sequencing were prepared using the Ion Chef System and Ion 540 Chip Kit followed by sequencing with the Ion S5 XL System (Thermo Fisher Scientific).

### Data analysis

Sequence data were subjected to a primary analysis using the AmpliSeq RNA plug-in of Ion Torrent Suite Software Plugins (Thermo Fisher Scientific). The obtained read-count data were then analyzed using R (3.6.1)^44^ and RStudio (1.1.442). For data cleaning, samples with percentage of genes detected (Targets Detected) values greater than 20% (calculated from the AmpliSeq RNA plug-in) were selected, and genes with non-zero read counts in more than 90% of the samples were selected. Normalized counts were obtained from read-count data by normalization with DESeq2 R package (Bioconductor)^45^. For the principal component analysis, normalized counts were transformed into variance-stabilizing transformation and analyzed in the top 1000 genes with high values of variance. For the extraction of DEGs, the likelihood ratio test using normalized counts was performed with *p*-value < 0.05 as a threshold value. Benjamini and Hochberg’s FDR < 0.05 or 0.25 as threshold values were used for adjustment of multiple testing. For the GO analysis, DEGs were used in DAVID^22,23^ to extract GO terms of biological process (Category: GOTERM_BP_DIRECT) and KEGG pathway (Category: KEGG_PATHWAY)^20,21^. The STRING database was then used to analyze protein–protein interactions^26^, and the basic settings were changed as follows: minimum required interaction score = low confidence (0.150). The plots were generated using the ggplot2^19^, gplots^24^, and ggbeeswarm^27^ R packages in combination with the reshape2^46^ and dplyr^47^ R packages.

### Machine learning

The first and second cohort data were combined into a single data set, and data cleaning and normalization were performed as described in the Data analysis section. Next, the combined data were split into 81 training and validation samples and 35 test samples. We used Python (3.7.4) and scikit-learn (0.22.2) in the training models. Rather than using all of the normalized expression levels, gene expression data with a detection rate *d_g* that was higher than the threshold *t* were used for training. The detection rate *d_g* is shown in Eq. (1), where for the gene *g*, *m* is the lower bound of the read count, *N_{g, m}* is the number of samples in which the read count of *g* is larger than *m*, and *N* is the number of samples.

1 $$ d\_g \, = \, N\_\left\{ {g, \, m} \right\}/N. $$

The expression levels in selected genes were then converted to rank values in descending order by samples, and the rank values were normalized to [0, 1] by gene. The rank normalized expression level, age, and sex were used as inputs for modeling. We used ERT, which suppresses overfitting compared with the Random Forest method^28^. For the training of models, the threshold of gene detection rate *t*, minimum of read count *m_r*, maximum depth of ERT, maximum number of features of ERT, minimum samples leaf of ERT, minimum samples split of ERT, and number of estimators of ERT were selected using tenfold cross-validation on the training and validation data. We used Optuna (1.3.0) to select parameters^48^. Using the chosen hyperparameters, we trained the models five times with different seeds, and then evaluated them with test data.

## Supplementary Information

Supplementary Information.

## Data Availability

The data produced from this study, including the clinical characteristics and scales and the transcriptome data, can be requested in Microsoft Excel format from the corresponding author. The code and data used for differential expression analysis and machine learning are available at GitHub (https://github.com/sssSSLp/PD_2021).
