# Supplementary material for: Non-invasive diagnostic tool for Parkinson’s disease by sebum RNA profile with machine learning
Source: Sci Rep. 2021 Sep 20;11:18550. doi: 10.1038/s41598-021-98423-9 (PMC8452747; doi:10.1038/s41598-021-98423-9)
Supplement: Supplementary file 1 — Supplementary Information. [file 41598_2021_98423_MOESM1_ESM.pdf]

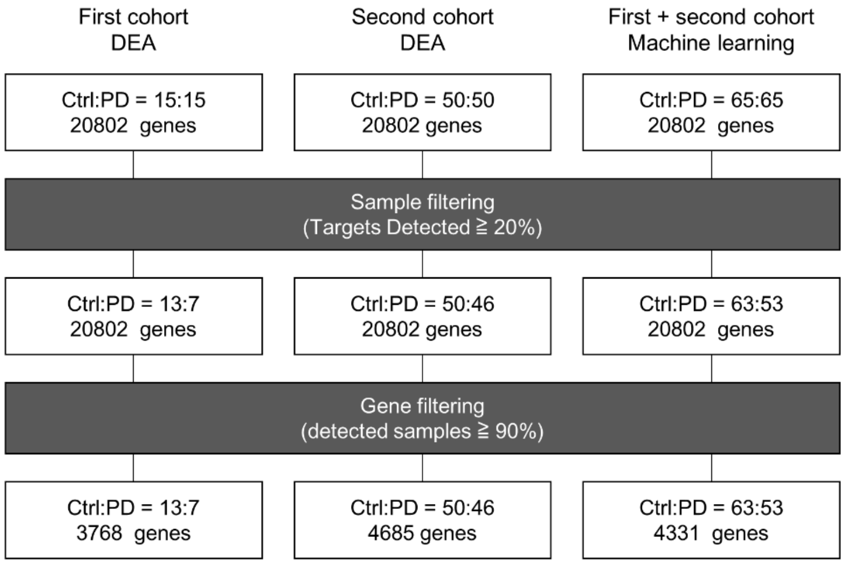

Supplementary Fig. S1. Data cleaning for the SSL-RNA analysis.

Cutoff criteria of the samples and genes. In the first cohort, 20 samples and 3768 genes were selected, and 96 samples and 4685 genes were selected in the second cohort. For machine learning, both cohorts were combined, and 116 samples and 4331 genes passed. Abbreviations: Ctrl = healthy controls; DEA = differential expression analysis; PD = Parkinson’s disease.

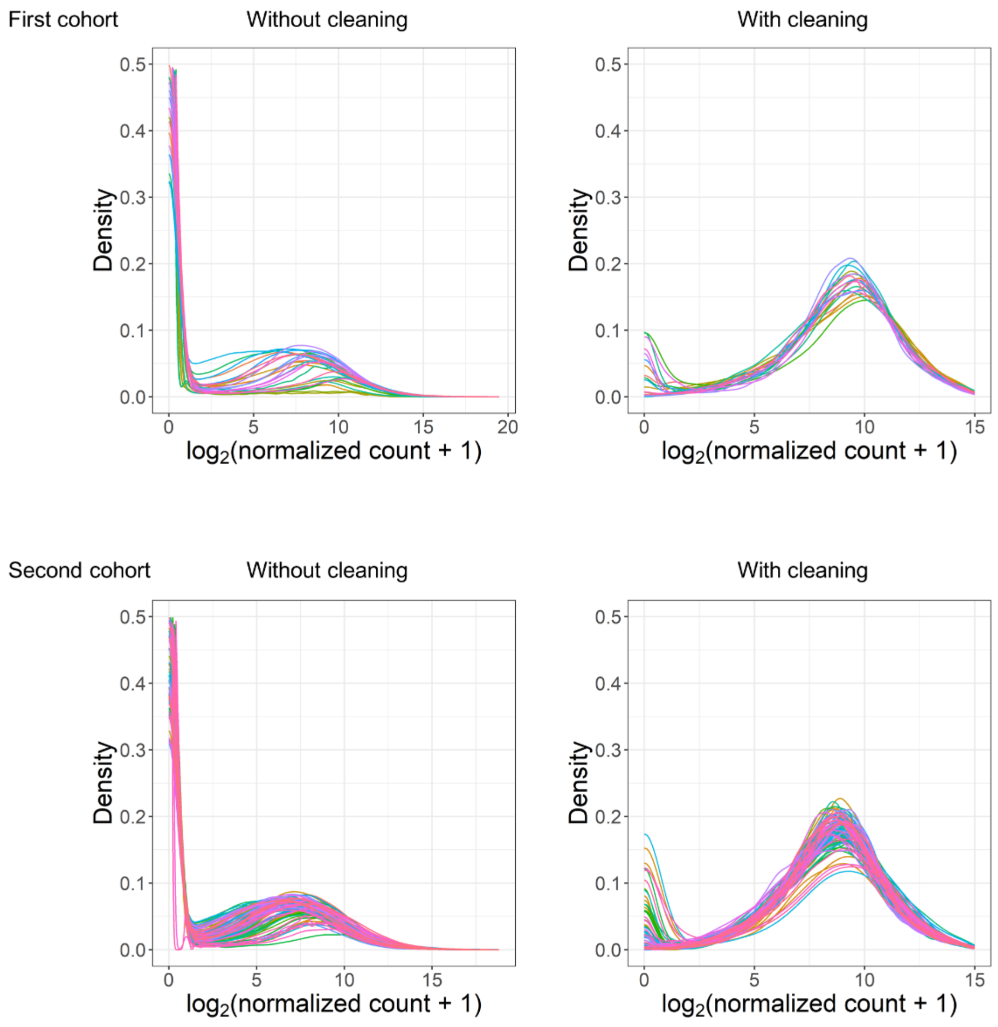

Supplementary Fig. S2. Effects of data cleaning to normalization.

Probability density distribution of the log2-transformed normalized count + 1, with or without data cleaning. Each plot represents the distribution of SSL-RNA expression levels in each sample. The uniformity of distribution was improved with data cleaning.

a

	Female and male		Male only	
	Healthy control	PD	Healthy control	PD
Number	13	7	6	6
Female : Male	7 : 6	1 : 6	0 : 6	0 : 6
Age (mean ± SD)	61.1 ± 11.7	58.4 ± 10.1	56.8 ± 10.1	56.3 ± 9.34
H&Y (cases)	(NA)	I (7), II (0)	(NA)	I (6)
H&Y (mean ± SD)	(NA)	1.00 ± 0	(NA)	1.00 ± 0
Duration (year, mean ± SD)	(NA)	0.643 ± 0.639	(NA)	0.667 ± 0.687
De novo patients (cases)	(NA)	7	(NA)	6

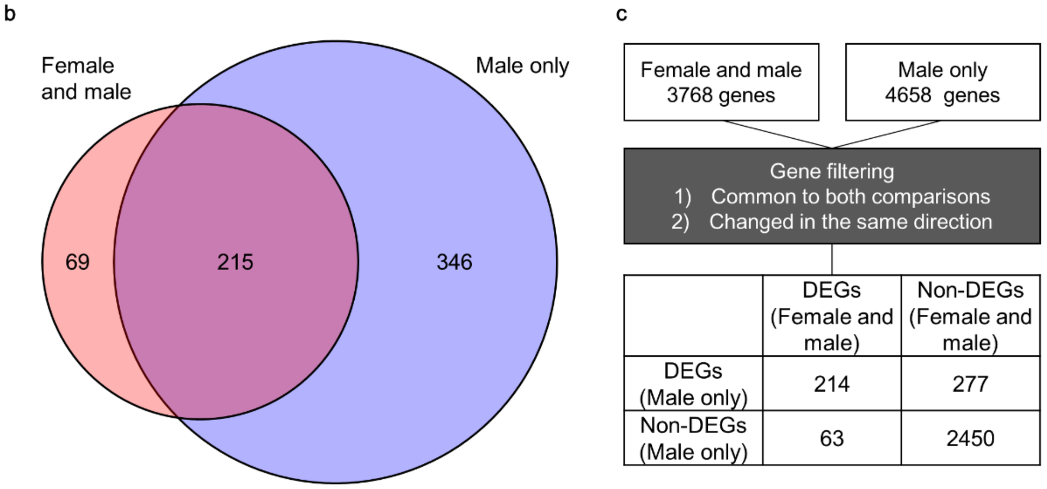

Supplementary Fig. S3. Effect of the female samples on differential expression analysis in the first cohort.

(a) Characteristics of male only and female and male groups. (b) Venn diagram of DEGs in both groups. (c) The overlap between male and female and male groups. Genes were filtered before comparison. The overlap of DEGs was tested by Fisher's exact test ($p < 0.01$). Abbreviations: SD = standard deviation; H&Y = Hoehn and Yahr stage; NA = not available; PD = Parkinson's disease; DEG = differentially expressed genes.

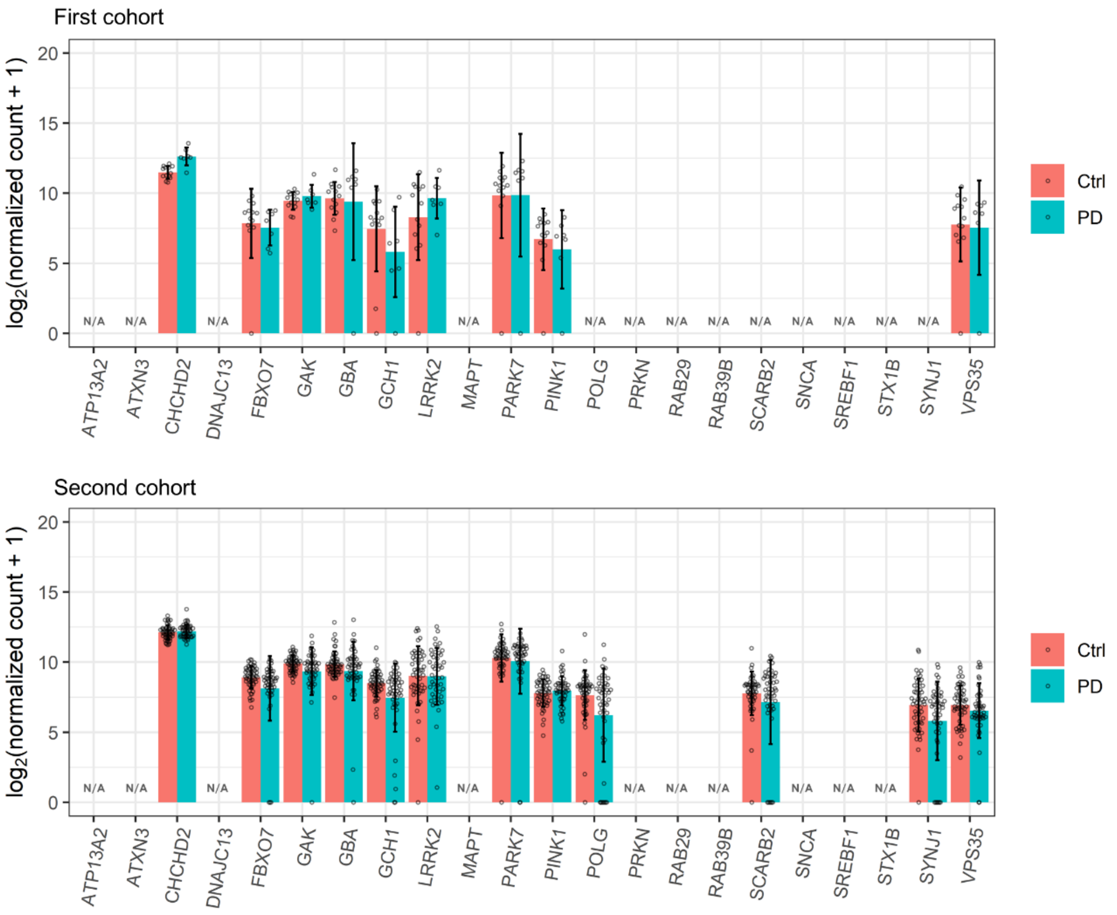

Supplementary Fig. S4. SSL-RNA expression changes in known PD genes.

Expression levels of known PD genes. Bar plots and error bars show the mean ± SD.

Beeswarm plots represent the gene expression levels in each subject. Abbreviations:

Ctrl = control; N/A = not available; PD = Parkinson's disease.

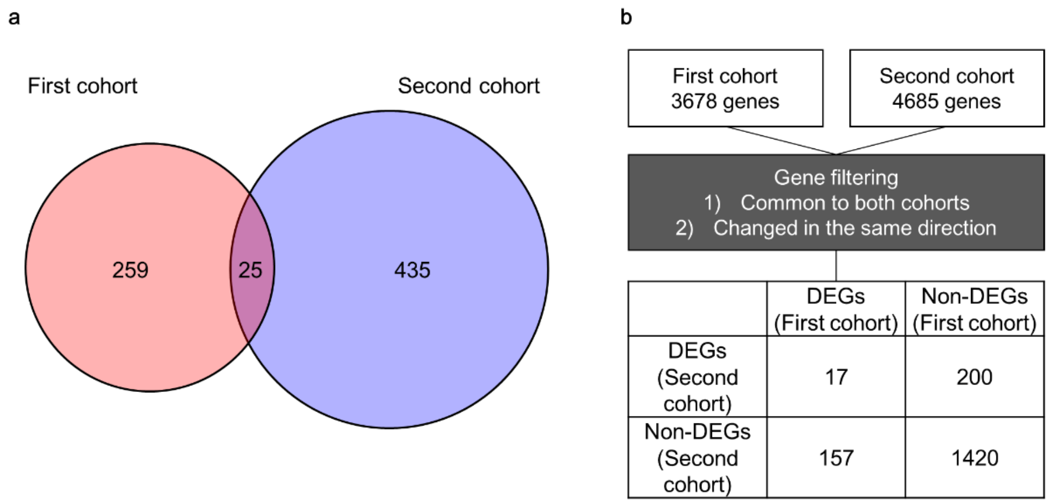

Supplementary Fig. S5. Overlap of DEGs between first and second cohorts.

(a) Venn diagram of DEGs in both cohorts. (b) Overlap between male and female and male groups. Genes were filtered before comparison. The overlap of DEGs was tested by Fisher's exact test (not significant). Abbreviations: DEG = differentially expressed genes.

Supplementary Table S1. Characteristics of participants after data cleaning.

	First cohort		Second cohort	
	Healthy control	PD	Healthy control	PD
Number	13	7	50	46
Female : Male	7 : 6	1 : 6	0 : 50	0 : 46
Age (mean ± SD)	61.1 ± 11.7	58.4 ± 10.1	66.8 ± 9.08	64.2 ± 10.9
H&Y (cases)	(NA)	I (7), II (0)	(NA)	0 (2), I (17), II (27)
H&Y (mean ± SD)	(NA)	1.00 ± 0	(NA)	1.54 ± 0.579
Duration (year, mean ± SD)	(NA)	0.643 ± 0.639	(NA)	5.67 ± 4.35
De novo patients (cases)	(NA)	7	(NA)	7

Abbreviations: NA = not available; SD = standard deviation.

Supplementary Table S2. GO terms and KEGG pathways obtained by DEGs with an FDR threshold of less than 0.05.

Cohort	Regulation	Category	Term	Count (%)	*p*-value	FDR
First	DOWN	GOTERM_BP_DIRECT	GO:0000184~nuclear-transcribed mRNA catabolic process, nonsense-mediated decay	7 (8.86)	1.29E-05	0.00677
		GOTERM_BP_DIRECT	GO:0006614~SRP-dependent cotranslational protein targeting to membrane	6 (7.59)	5.49E-05	0.0144
		GOTERM_BP_DIRECT	GO:0019083~viral transcription	6 (7.59)	0.000126	0.0221
		KEGG_PATHWAY	hsa03010:Ribosome	7 (8.86)	0.000136	0.0116
		GOTERM_BP_DIRECT	GO:0006413~translational initiation	6 (7.59)	0.000323	0.0424
		GOTERM_BP_DIRECT	GO:0006412~translation	7 (8.86)	0.000807	0.0848
		GOTERM_BP_DIRECT	GO:0098609~cell-cell adhesion	7 (8.86)	0.00115	0.101
		GOTERM_BP_DIRECT	GO:0006364~rRNA processing	6 (7.59)	0.00239	0.179
		KEGG_PATHWAY	hsa04144:Endocytosis	7 (8.86)	0.00279	0.119
		GOTERM_BP_DIRECT	GO:0000027~ribosomal large subunit assembly	3 (3.80)	0.00371	0.244
		GOTERM_BP_DIRECT	GO:0035088~establishment or maintenance of apical/basal cell polarity	2 (2.53)	0.0216	1.00
		GOTERM_BP_DIRECT	GO:0006897~endocytosis	4 (5.06)	0.0227	1.00
		KEGG_PATHWAY	hsa05203:Viral carcinogenesis	5 (6.33)	0.0328	0.930
		GOTERM_BP_DIRECT	GO:0016197~endosomal transport	3 (3.80)	0.0334	1.00
		GOTERM_BP_DIRECT	GO:1904903~ESCRT III complex disassembly	2 (2.53)	0.0426	1.00
		GOTERM_BP_DIRECT	GO:1902188~positive regulation of viral release from host cell	2 (2.53)	0.0468	1.00

	GOTERM_BP_DIRECT	GO:1901653~cellular response to peptide	2 (2.53)	0.0468	1.00
	GOTERM_BP_DIRECT	GO:0048013~ephrin receptor signaling pathway	3 (3.80)	0.0539	1.00
	KEGG_PATHWAY	hsa05211:Renal cell carcinoma	3 (3.80)	0.0585	1.00
	GOTERM_BP_DIRECT	GO:1901673~regulation of mitotic spindle assembly	2 (2.53)	0.0592	1.00
	GOTERM_BP_DIRECT	GO:0007034~vacuolar transport	2 (2.53)	0.0674	1.00
	GOTERM_BP_DIRECT	GO:0097194~execution phase of apoptosis	2 (2.53)	0.0674	1.00
	GOTERM_BP_DIRECT	GO:0000920~cell separation after cytokinesis	2 (2.53)	0.0755	1.00
	GOTERM_BP_DIRECT	GO:0010824~regulation of centrosome duplication	2 (2.53)	0.0795	1.00
	GOTERM_BP_DIRECT	GO:0051493~regulation of cytoskeleton organization	2 (2.53)	0.0795	1.00
	GOTERM_BP_DIRECT	GO:0006886~intracellular protein transport	4 (5.06)	0.0834	1.00
	GOTERM_BP_DIRECT	GO:0050850~positive regulation of calcium-mediated signaling	2 (2.53)	0.0835	1.00
	KEGG_PATHWAY	hsa05132:Salmonella infection	3 (3.80)	0.0872	1.00
	GOTERM_BP_DIRECT	GO:0006921~cellular component disassembly involved in execution phase of apoptosis	2 (2.53)	0.0875	1.00
	GOTERM_BP_DIRECT	GO:0039702~viral budding via host ESCRT complex	2 (2.53)	0.0915	1.00
	GOTERM_BP_DIRECT	GO:0007264~small GTPase mediated signal transduction	4 (5.06)	0.0917	1.00
	GOTERM_BP_DIRECT	GO:0015031~protein transport	5 (6.33)	0.0930	1.00
	KEGG_PATHWAY	hsa05168:Herpes simplex infection	4 (5.06)	0.0947	1.00
	KEGG_PATHWAY	hsa04062:Chemokine signaling pathway	4 (5.06)	0.0983	1.00
	GOTERM_BP_DIRECT	GO:0043547~positive regulation of GTPase activity	6 (7.59)	0.0991	1.00
UP	KEGG_PATHWAY	hsa00190:Oxidative phosphorylation	2 (13.3)	0.0751	0.321

		KEGG_PATHWAY	hsa05012:Parkinson's disease	2 (13.3)	0.0801	0.321
		GOTERM_BP_DIRECT	GO:0006511~ubiquitin-dependent protein catabolic process	2 (13.3)	0.0934	1.00
		KEGG_PATHWAY	hsa05010:Alzheimer's disease	2 (13.3)	0.0942	0.321
Second	DOWN	GOTERM_BP_DIRECT	GO:0006915~apoptotic process	11 (22.5)	1.96E-06	0.000864
		GOTERM_BP_DIRECT	GO:0006954~inflammatory response	8 (16.3)	6.12E-05	0.0135
		GOTERM_BP_DIRECT	GO:0043066~negative regulation of apoptotic process	6 (12.2)	0.00716	1.00
		KEGG_PATHWAY	hsa04380:Osteoclast differentiation	4 (8.16)	0.00798	0.566
		GOTERM_BP_DIRECT	GO:0048146~positive regulation of fibroblast proliferation	3 (6.12)	0.00920	1.00
		GOTERM_BP_DIRECT	GO:0045944~positive regulation of transcription from RNA polymerase II promoter	8 (16.3)	0.0147	1.00
		GOTERM_BP_DIRECT	GO:0071347~cellular response to interleukin-1	3 (6.12)	0.0155	1.00
		GOTERM_BP_DIRECT	GO:0016236~macroautophagy	3 (6.12)	0.0177	1.00
		KEGG_PATHWAY	hsa05323:Rheumatoid arthritis	3 (6.12)	0.0317	1.00
		GOTERM_BP_DIRECT	GO:0071356~cellular response to tumor necrosis factor	3 (6.12)	0.0351	1.00
		GOTERM_BP_DIRECT	GO:1900745~positive regulation of p38MAPK cascade	2 (4.08)	0.0420	1.00
		GOTERM_BP_DIRECT	GO:0051412~response to corticosterone	2 (4.08)	0.0472	1.00
		GOTERM_BP_DIRECT	GO:0051092~positive regulation of NF-kappaB transcription factor activity	3 (6.12)	0.0494	1.00
		GOTERM_BP_DIRECT	GO:0010629~negative regulation of gene expression	3 (6.12)	0.0521	1.00
		GOTERM_BP_DIRECT	GO:0007050~cell cycle arrest	3 (6.12)	0.0548	1.00
		KEGG_PATHWAY	hsa04068:FoxO signaling pathway	3 (6.12)	0.0675	1.00
		GOTERM_BP_DIRECT	GO:0048247~lymphocyte chemotaxis	2 (4.08)	0.0724	1.00

	GOTERM_BP_DIRECT	GO:2000379~positive regulation of reactive oxygen species metabolic process	2 (4.08)	0.0774	1.00
	GOTERM_BP_DIRECT	GO:0048167~regulation of synaptic plasticity	2 (4.08)	0.0824	1.00
	GOTERM_BP_DIRECT	GO:0031663~lipopolysaccharide-mediated signaling pathway	2 (4.08)	0.0824	1.00
	GOTERM_BP_DIRECT	GO:0030335~positive regulation of cell migration	3 (6.12)	0.0871	1.00
	GOTERM_BP_DIRECT	GO:0000422~mitophagy	2 (4.08)	0.0897	1.00
UP	GOTERM_BP_DIRECT	GO:0031424~keratinization	8 (19.1)	6.25E-12	1.11E-09
	GOTERM_BP_DIRECT	GO:0006413~translational initiation	9 (21.4)	3.12E-10	2.76E-08
	GOTERM_BP_DIRECT	GO:0006614~SRP-dependent cotranslational protein targeting to membrane	8 (19.1)	8.12E-10	4.79E-08
	KEGG_PATHWAY	hsa03010:Ribosome	8 (19.1)	1.57E-09	2.20E-08
	GOTERM_BP_DIRECT	GO:0019083~viral transcription	8 (19.1)	2.80E-09	1.24E-07
	GOTERM_BP_DIRECT	GO:0000184~nuclear-transcribed mRNA catabolic process, nonsense-mediated decay	8 (19.1)	4.29E-09	1.52E-07
	GOTERM_BP_DIRECT	GO:0018149~peptide cross-linking	6 (14.3)	5.78E-08	1.71E-06
	GOTERM_BP_DIRECT	GO:0006364~rRNA processing	8 (19.1)	2.46E-07	6.22E-06
	GOTERM_BP_DIRECT	GO:0030216~keratinocyte differentiation	6 (14.3)	4.85E-07	1.07E-05
	GOTERM_BP_DIRECT	GO:0006412~translation	8 (19.1)	7.61E-07	0.000015
	GOTERM_BP_DIRECT	GO:0008544~epidermis development	6 (14.3)	8.50E-07	0.000015
	GOTERM_BP_DIRECT	GO:0042254~ribosome biogenesis	4 (9.52)	5.65E-05	0.000909
	GOTERM_BP_DIRECT	GO:0002181~cytoplasmic translation	3 (7.14)	0.00123	0.0181
	GOTERM_BP_DIRECT	GO:0031069~hair follicle morphogenesis	3 (7.14)	0.00143	0.0195

GOTERM_BP_DIRECT	GO:0098609~cell-cell adhesion	4 (9.52)	0.0186	0.235
GOTERM_BP_DIRECT	GO:0045109~intermediate filament organization	2 (4.76)	0.0328	0.388
GOTERM_BP_DIRECT	GO:0045653~negative regulation of megakaryocyte differentiation	2 (4.76)	0.0369	0.408
GOTERM_BP_DIRECT	GO:0071385~cellular response to glucocorticoid stimulus	2 (4.76)	0.0409	0.426
GOTERM_BP_DIRECT	GO:0007010~cytoskeleton organization	3 (7.14)	0.0442	0.435
GOTERM_BP_DIRECT	GO:0016233~telomere capping	2 (4.76)	0.0469	0.437
GOTERM_BP_DIRECT	GO:0006336~DNA replication-independent nucleosome assembly	2 (4.76)	0.0528	0.462
GOTERM_BP_DIRECT	GO:0032200~telomere organization	2 (4.76)	0.0548	0.462
GOTERM_BP_DIRECT	GO:0006335~DNA replication-dependent nucleosome assembly	2 (4.76)	0.0646	0.520
GOTERM_BP_DIRECT	GO:0097192~extrinsic apoptotic signaling pathway in absence of ligand	2 (4.76)	0.0685	0.527
GOTERM_BP_DIRECT	GO:0006352~DNA-templated transcription, initiation	2 (4.76)	0.0724	0.527
GOTERM_BP_DIRECT	GO:0000183~chromatin silencing at rDNA	2 (4.76)	0.0744	0.527
GOTERM_BP_DIRECT	GO:0051290~protein heterotetramerization	2 (4.76)	0.0840	0.543
GOTERM_BP_DIRECT	GO:1904837~beta-catenin-TCF complex assembly	2 (4.76)	0.0859	0.543
GOTERM_BP_DIRECT	GO:0034080~CENP-A containing nucleosome assembly	2 (4.76)	0.0859	0.543
GOTERM_BP_DIRECT	GO:0045814~negative regulation of gene expression, epigenetic	2 (4.76)	0.0992	0.605

Enriched GO terms and KEGG pathways were yielded by DAVID (Category: GOTERM_BP_DIRECT and KEGG_PATHWAY) in each

cohort using the downregulated or upregulated DEGs with an FDR threshold of less than 0.05.

Supplementary Table S3. KEGG pathways obtained using the DEGs in each cohort.

Cohort	Regulation	Term	Count (%)	*p*-value	FDR
First	DOWN	hsa03010:Ribosome	13 (6.28)	1.21E-06	0.000180
		hsa04144:Endocytosis	15 (7.25)	2.13E-05	0.00159
		hsa05203:Viral carcinogenesis	11 (5.31)	0.00135	0.0670
		hsa05211:Renal cell carcinoma	6 (2.90)	0.00363	0.135
		hsa05168:Herpes simplex infection	9 (4.35)	0.00787	0.196
		hsa05130:Pathogenic Escherichia coli infection	5 (2.42)	0.00815	0.196
		hsa04670:Leukocyte transendothelial migration	7 (3.38)	0.00920	0.196
		hsa05323:Rheumatoid arthritis	6 (2.90)	0.0121	0.226
		hsa04141:Protein processing in endoplasmic reticulum	8 (3.86)	0.0167	0.251
		hsa03040:Spliceosome	7 (3.38)	0.0179	0.251
		hsa04068:FoxO signaling pathway	7 (3.38)	0.0185	0.251
		hsa04062:Chemokine signaling pathway	8 (3.86)	0.0266	0.331
		hsa04140:Regulation of autophagy	3 (1.45)	0.0580	0.664
		hsa05205:Proteoglycans in cancer	7 (3.38)	0.0940	0.961
	UP	hsa04932:Non-alcoholic fatty liver disease (NAFLD)	6 (8.11)	0.000794	0.0833
		hsa05016:Huntington's disease	6 (8.11)	0.00232	0.122
		hsa00190:Oxidative phosphorylation	4 (5.41)	0.0273	0.848
		hsa05012:Parkinson's disease	4 (5.41)	0.0323	0.848
		hsa05010:Alzheimer's disease	4 (5.41)	0.0493	0.920

		hsa04260:Cardiac muscle contraction	3 (4.05)	0.0526	0.920
		hsa04066:HIF-1 signaling pathway	3 (4.05)	0.0811	1.00
Second	DOWN	hsa04062:Chemokine signaling pathway	15 (4.89)	2.08E-05	0.00380
		hsa04722:Neurotrophin signaling pathway	11 (3.58)	0.000144	0.0131
		hsa04380:Osteoclast differentiation	11 (3.58)	0.000296	0.0180
		hsa04142:Lysosome	10 (3.26)	0.000720	0.0312
		hsa05203:Viral carcinogenesis	13 (4.23)	0.000853	0.0312
		hsa04144:Endocytosis	14 (4.56)	0.00111	0.0340
		hsa04064:NF-kappa B signaling pathway	8 (2.61)	0.00180	0.0470
		hsa04662:B cell receptor signaling pathway	7 (2.28)	0.00258	0.0548
		hsa04920:Adipocytokine signaling pathway	7 (2.28)	0.00277	0.0548
		hsa05152:Tuberculosis	11 (3.58)	0.00300	0.0548
		hsa04010:MAPK signaling pathway	13 (4.23)	0.00497	0.0827
		hsa05169:Epstein-Barr virus infection	8 (2.61)	0.0115	0.176
		hsa05220:Chronic myeloid leukemia	6 (1.95)	0.0149	0.202
		hsa04660:T cell receptor signaling pathway	7 (2.28)	0.0154	0.202
		hsa05168:Herpes simplex infection	9 (2.93)	0.0316	0.365
		hsa04145:Phagosome	8 (2.61)	0.0319	0.365
		hsa04210:Apoptosis	5 (1.63)	0.0361	0.389
		hsa05131:Shigellosis	5 (1.63)	0.0399	0.406
		hsa03320:PPAR signaling pathway	5 (1.63)	0.0460	0.443
		hsa05205:Proteoglycans in cancer	9 (2.93)	0.0491	0.450

	hsa04060:Cytokine-cytokine receptor interaction	10 (3.26)	0.0571	0.497
	hsa04620:Toll-like receptor signaling pathway	6 (1.95)	0.0629	0.523
	hsa04931:Insulin resistance	6 (1.95)	0.0671	0.533
	hsa05166:HTLV-I infection	10 (3.26)	0.0712	0.533
	hsa05161:Hepatitis B	7 (2.28)	0.0729	0.533
	hsa05132:Salmonella infection	5 (1.63)	0.0866	0.581
	hsa04666:Fc gamma R-mediated phagocytosis	5 (1.63)	0.0896	0.581
	hsa05222:Small cell lung cancer	5 (1.63)	0.0926	0.581
	hsa04071:Sphingolipid signaling pathway	6 (1.95)	0.0952	0.581
	hsa05134:Legionellosis	4 (1.3)	0.0952	0.581
	hsa05200:Pathways in cancer	13 (4.23)	0.0991	0.585
UP	hsa03010:Ribosome	37 (24.5)	1.04E-42	9.40E-41
	hsa00190:Oxidative phosphorylation	13 (8.61)	1.79E-08	8.07E-07
	hsa05012:Parkinson's disease	12 (7.95)	3.58E-07	1.07E-05
	hsa05010:Alzheimer's disease	12 (7.95)	1.93E-06	4.33E-05
	hsa05016:Huntington's disease	12 (7.95)	7.06E-06	0.000127
	hsa04932:Non-alcoholic fatty liver disease (NAFLD)	9 (5.96)	0.000239	0.00358
	hsa04260:Cardiac muscle contraction	4 (2.65)	0.0497	0.639

Enriched KEGG pathways were yielded by DAVID (Category: KEGG_PATHWAY) in each cohort using downregulated or upregulated DEGs.

Supplementary Table S4. GO terms of biological process obtained using the DEGs in each cohort.

Cohort	Regulation	Term	Count (%)	*p*-value	FDR
First	DOWN	GO:0000184~nuclear-transcribed mRNA catabolic process, nonsense-mediated decay	12 (5.80)	9.53E-08	5.99E-05
		GO:0006614~SRP-dependent cotranslational protein targeting to membrane	11 (5.31)	9.75E-08	5.99E-05
		GO:0006413~translational initiation	12 (5.80)	4.02E-07	0.000158
		GO:0019083~viral transcription	11 (5.31)	5.13E-07	0.000158
		GO:0006364~rRNA processing	13 (6.28)	5.37E-06	0.00132
		GO:0006412~translation	13 (6.28)	0.0000286	0.00585
		GO:0015031~protein transport	15 (7.25)	0.000149	0.0262
		GO:1902188~positive regulation of viral release from host cell	4 (1.93)	0.000213	0.0327
		GO:0061028~establishment of endothelial barrier	4 (1.93)	0.000695	0.0920
		GO:0006914~autophagy	8 (3.86)	0.000749	0.0920
		GO:0000398~mRNA splicing, via spliceosome	10 (4.83)	0.000907	0.100
		GO:0098609~cell-cell adhesion	11 (5.31)	0.000977	0.100
		GO:0035088~establishment or maintenance of apical/basal cell polarity	3 (1.45)	0.00122	0.115
		GO:0000027~ribosomal large subunit assembly	4 (1.93)	0.00158	0.139
		GO:0039702~viral budding via host ESCRT complex	4 (1.93)	0.00182	0.149
		GO:0010467~gene expression	5 (2.42)	0.00202	0.155

GO:0019058~viral life cycle	4 (1.93)	0.00493	0.336
GO:0036258~multivesicular body assembly	4 (1.93)	0.00493	0.336
GO:0006366~transcription from RNA polymerase II promoter	14 (6.76)	0.00522	0.337
GO:0016197~endosomal transport	5 (2.42)	0.00639	0.389
GO:0016032~viral process	10 (4.83)	0.00666	0.389
GO:0042059~negative regulation of epidermal growth factor receptor signaling pathway	4 (1.93)	0.00752	0.420
GO:0010628~positive regulation of gene expression	9 (4.35)	0.00948	0.506
GO:1901673~regulation of mitotic spindle assembly	3 (1.45)	0.0104	0.531
GO:0045454~cell redox homeostasis	5 (2.42)	0.0109	0.536
GO:0030033~microvillus assembly	3 (1.45)	0.0152	0.717
GO:0048013~ephrin receptor signaling pathway	5 (2.42)	0.0159	0.721
GO:0000920~cell separation after cytokinesis	3 (1.45)	0.0170	0.739
GO:0021762~substantia nigra development	4 (1.93)	0.0175	0.739
GO:0010824~regulation of centrosome duplication	3 (1.45)	0.0188	0.745
GO:0006895~Golgi to endosome transport	3 (1.45)	0.0188	0.745
GO:0007173~epidermal growth factor receptor signaling pathway	4 (1.93)	0.0248	0.925
GO:0034314~Arp2/3 complex-mediated actin nucleation	3 (1.45)	0.0249	0.925
GO:0006367~transcription initiation from RNA polymerase II promoter	6 (2.90)	0.0282	0.994
GO:0097352~autophagosome maturation	3 (1.45)	0.0316	0.994

GO:0002181~cytoplasmic translation	3 (1.45)	0.0316	0.994
GO:0000122~negative regulation of transcription from RNA polymerase II promoter	15 (7.25)	0.0319	0.994
GO:0061763~multivesicular body-lysosome fusion	2 (0.966)	0.0332	0.994
GO:0018076~N-terminal peptidyl-lysine acetylation	2 (0.966)	0.0332	0.994
GO:0006997~nucleus organization	3 (1.45)	0.0340	0.994
GO:0034599~cellular response to oxidative stress	4 (1.93)	0.0350	0.994
GO:0070979~protein K11-linked ubiquitination	3 (1.45)	0.0364	0.994
GO:0051291~protein heterooligomerization	4 (1.93)	0.0393	0.994
GO:0045766~positive regulation of angiogenesis	5 (2.42)	0.0403	0.994
GO:0006461~protein complex assembly	5 (2.42)	0.0414	0.994
GO:0097421~liver regeneration	3 (1.45)	0.0415	0.994
GO:2000301~negative regulation of synaptic vesicle exocytosis	2 (0.966)	0.0440	0.994
GO:0018393~internal peptidyl-lysine acetylation	2 (0.966)	0.0440	0.994
GO:0097411~hypoxia-inducible factor-1alpha signaling pathway	2 (0.966)	0.0440	0.994
GO:1901888~regulation of cell junction assembly	2 (0.966)	0.0440	0.994
GO:0006990~positive regulation of transcription from RNA polymerase II promoter involved in unfolded protein response	2 (0.966)	0.0440	0.994
GO:0097368~establishment of Sertoli cell barrier	2 (0.966)	0.0440	0.994
GO:0033522~histone H2A ubiquitination	2 (0.966)	0.0440	0.994
GO:0048010~vascular endothelial growth factor receptor signaling pathway	4 (1.93)	0.0470	0.994

GO:0045444~fat cell differentiation	4 (1.93)	0.0486	0.994
GO:0006886~intracellular protein transport	7 (3.38)	0.0500	0.994
GO:0006283~transcription-coupled nucleotide-excision repair	4 (1.93)	0.0502	0.994
GO:0034976~response to endoplasmic reticulum stress	4 (1.93)	0.0519	0.994
GO:0006511~ubiquitin-dependent protein catabolic process	6 (2.90)	0.0541	0.994
GO:0038096~Fc-gamma receptor signaling pathway involved in phagocytosis	5 (2.42)	0.0545	0.994
GO:0022614~membrane to membrane docking	2 (0.966)	0.0547	0.994
GO:1901985~positive regulation of protein acetylation	2 (0.966)	0.0547	0.994
GO:0006658~phosphatidylserine metabolic process	2 (0.966)	0.0547	0.994
GO:0008286~insulin receptor signaling pathway	4 (1.93)	0.0571	0.994
GO:0007264~small GTPase mediated signal transduction	7 (3.38)	0.0587	0.994
GO:0030177~positive regulation of Wnt signaling pathway	3 (1.45)	0.0613	0.994
GO:0007080~mitotic metaphase plate congression	3 (1.45)	0.0643	0.994
GO:0008543~fibroblast growth factor receptor signaling pathway	4 (1.93)	0.0644	0.994
GO:0050792~regulation of viral process	2 (0.966)	0.0653	0.994
GO:0009790~embryo development	3 (1.45)	0.0674	0.994
GO:0006605~protein targeting	3 (1.45)	0.0705	0.994
GO:0036089~cleavage furrow formation	2 (0.966)	0.0758	0.994
GO:0071312~cellular response to alkaloid	2 (0.966)	0.0758	0.994
GO:0009411~response to UV	3 (1.45)	0.0802	0.994

	GO:0051496~positive regulation of stress fiber assembly	3 (1.45)	0.0802	0.994
	GO:0090002~establishment of protein localization to plasma membrane	3 (1.45)	0.0802	0.994
	GO:0031623~receptor internalization	3 (1.45)	0.0835	0.994
	GO:0043966~histone H3 acetylation	3 (1.45)	0.0835	0.994
	GO:0030838~positive regulation of actin filament polymerization	3 (1.45)	0.0903	0.994
	GO:0071456~cellular response to hypoxia	4 (1.93)	0.0930	0.994
	GO:0015825~L-serine transport	2 (0.966)	0.0964	0.994
	GO:0006473~protein acetylation	2 (0.966)	0.0964	0.994
	GO:1904754~positive regulation of vascular associated smooth muscle cell migration	2 (0.966)	0.0964	0.994
	GO:0032956~regulation of actin cytoskeleton organization	3 (1.45)	0.0972	0.994
	GO:0070936~protein K48-linked ubiquitination	3 (1.45)	0.0972	0.994
	GO:0045860~positive regulation of protein kinase activity	3 (1.45)	0.0972	0.994
UP	GO:0043434~response to peptide hormone	4 (5.41)	0.000543	0.304
	GO:0043066~negative regulation of apoptotic process	8 (10.8)	0.00126	0.304
	GO:0006122~mitochondrial electron transport, ubiquinol to cytochrome c	3 (4.05)	0.00132	0.304
	GO:0043488~regulation of mRNA stability	4 (5.41)	0.00623	1.00
	GO:0031394~positive regulation of prostaglandin biosynthetic process	2 (0.966)	0.0145	1.00
	GO:1902600~hydrogen ion transmembrane transport	3 (4.05)	0.0207	1.00

GO:0010042~response to manganese ion	2 (2.7)	0.0216	1.00
GO:0032496~response to lipopolysaccharide	4 (5.41)	0.0218	1.00
GO:0032436~positive regulation of proteasomal ubiquitin-dependent protein catabolic process	3 (4.05)	0.0227	1.00
GO:0031340~positive regulation of vesicle fusion	2 (0.966)	0.0252	1.00
GO:0090050~positive regulation of cell migration involved in sprouting angiogenesis	2 (0.966)	0.0252	1.00
GO:0042060~wound healing	3 (4.05)	0.0342	1.00
GO:0006928~movement of cell or subcellular component	3 (4.05)	0.0390	1.00
GO:0032355~response to estradiol	3 (4.05)	0.0432	1.00
GO:0008283~cell proliferation	5 (6.76)	0.0439	1.00
GO:0006119~oxidative phosphorylation	2 (0.966)	0.0462	1.00
GO:0071456~cellular response to hypoxia	3 (4.05)	0.0476	1.00
GO:0042633~hair cycle	2 (0.966)	0.0531	1.00
GO:0030224~monocyte differentiation	2 (0.966)	0.0600	1.00
GO:0060749~mammary gland alveolus development	2 (0.966)	0.0600	1.00
GO:0071356~cellular response to tumor necrosis factor	3 (4.05)	0.0606	1.00
GO:0030225~macrophage differentiation	2 (0.966)	0.0634	1.00
GO:0033280~response to vitamin D	2 (0.966)	0.0634	1.00
GO:0051493~regulation of cytoskeleton organization	2 (0.966)	0.0668	1.00
GO:0071385~cellular response to glucocorticoid stimulus	2 (0.966)	0.0702	1.00
GO:0046697~decidualization	2 (0.966)	0.0702	1.00

		GO:0045087~innate immune response	5 (6.76)	0.0708	1.00
		GO:0010951~negative regulation of endopeptidase activity	3 (4.05)	0.0716	1.00
		GO:0051894~positive regulation of focal adhesion assembly	2 (0.966)	0.0736	1.00
		GO:0098609~cell-cell adhesion	4 (5.41)	0.0756	1.00
		GO:0007166~cell surface receptor signaling pathway	4 (5.41)	0.0776	1.00
		GO:0030036~actin cytoskeleton organization	3 (4.05)	0.0810	1.00
		GO:0030099~myeloid cell differentiation	2 (0.966)	0.0837	1.00
		GO:0072661~protein targeting to plasma membrane	2 (0.966)	0.0903	1.00
		GO:0008360~regulation of cell shape	3 (4.05)	0.0919	1.00
		GO:0032007~negative regulation of TOR signaling	2 (0.966)	0.0970	1.00
		GO:0042493~response to drug	4 (5.41)	0.0985	1.00
Second	DOWN	GO:0006954~inflammatory response	21 (6.84)	7.44E-06	0.0126
		GO:0098609~cell-cell adhesion	16 (5.21)	6.14E-05	0.0294
		GO:0007264~small GTPase mediated signal transduction	15 (4.89)	8.02E-05	0.0294
		GO:0043123~positive regulation of I-kappaB kinase/NF-kappaB signaling	12 (3.91)	9.44E-05	0.0294
		GO:0006915~apoptotic process	24 (7.82)	0.000102	0.0294
		GO:0071356~cellular response to tumor necrosis factor	10 (3.26)	0.000104	0.0294
		GO:0032088~negative regulation of NF-kappaB transcription factor activity	8 (2.61)	0.000194	0.0468
		GO:0007165~signal transduction	37 (12.1)	0.000312	0.0660
		GO:0051092~positive regulation of NF-kappaB transcription	10 (3.26)	0.000436	0.0819

factor activity			
GO:0071222~cellular response to lipopolysaccharide	9 (2.93)	0.000670	0.113
GO:0051591~response to cAMP	6 (1.95)	0.00104	0.160
GO:0045944~positive regulation of transcription from RNA polymerase II promoter	31 (10.1)	0.00125	0.175
GO:0032496~response to lipopolysaccharide	10 (3.26)	0.00193	0.239
GO:0043066~negative regulation of apoptotic process	18 (5.86)	0.00198	0.239
GO:0050731~positive regulation of peptidyl-tyrosine phosphorylation	7 (2.28)	0.00265	0.299
GO:0006968~cellular defense response	6 (1.95)	0.00394	0.416
GO:0042127~regulation of cell proliferation	10 (3.26)	0.00431	0.429
GO:0006952~defense response	6 (1.95)	0.00549	0.467
GO:0010886~positive regulation of cholesterol storage	3 (0.977)	0.00566	0.467
GO:0038066~p38MAPK cascade	3 (0.977)	0.00566	0.467
GO:0007265~Ras protein signal transduction	6 (1.95)	0.00661	0.467
GO:0035335~peptidyl-tyrosine dephosphorylation	7 (2.28)	0.00670	0.467
GO:0071347~cellular response to interleukin-1	6 (1.95)	0.00701	0.467
GO:0070098~chemokine-mediated signaling pathway	6 (1.95)	0.00701	0.467
GO:0034383~low-density lipoprotein particle clearance	3 (0.977)	0.00746	0.467
GO:0061179~negative regulation of insulin secretion involved in cellular response to glucose stimulus	3 (0.977)	0.00746	0.467
GO:0048227~plasma membrane to endosome transport	3 (0.977)	0.00746	0.467

GO:0051056~regulation of small GTPase mediated signal transduction	8 (2.61)	0.00780	0.471
GO:0000188~inactivation of MAPK activity	4 (1.30)	0.00836	0.487
GO:0016236~macroautophagy	6 (1.95)	0.00930	0.509
GO:0015992~proton transport	5 (1.63)	0.00934	0.509
GO:0007266~Rho protein signal transduction	5 (1.63)	0.0100	0.529
GO:0006468~protein phosphorylation	16 (5.21)	0.0111	0.566
GO:0034097~response to cytokine	5 (1.63)	0.0115	0.571
GO:0016311~dephosphorylation	6 (1.95)	0.0153	0.739
GO:0061158~3'-UTR-mediated mRNA destabilization	3 (0.977)	0.0168	0.767
GO:0001706~endoderm formation	3 (0.977)	0.0168	0.767
GO:0015031~protein transport	14 (4.56)	0.0172	0.767
GO:0043922~negative regulation by host of viral transcription	3 (0.977)	0.0197	0.811
GO:0071850~mitotic cell cycle arrest	3 (0.977)	0.0197	0.811
GO:0006950~response to stress	5 (1.63)	0.0197	0.811
GO:0050727~regulation of inflammatory response	5 (1.63)	0.0219	0.871
GO:0043407~negative regulation of MAP kinase activity	4 (1.30)	0.0227	0.871
GO:0031929~TOR signaling	3 (0.977)	0.0227	0.871
GO:0006974~cellular response to DNA damage stimulus	9 (2.93)	0.0254	0.921
GO:0030593~neutrophil chemotaxis	5 (1.63)	0.0255	0.921
GO:0006914~autophagy	7 (2.28)	0.0256	0.921

GO:0006469~negative regulation of protein kinase activity	6 (1.95)	0.0264	0.929
GO:0043124~negative regulation of I-kappaB kinase/NF-kappaB signaling	4 (1.30)	0.0299	0.996
GO:0008654~phospholipid biosynthetic process	4 (1.30)	0.0299	0.996
GO:0008360~regulation of cell shape	7 (2.28)	0.0318	0.996
GO:0050860~negative regulation of T cell receptor signaling pathway	3 (0.977)	0.0328	0.996
GO:1904582~positive regulation of intracellular mRNA localization	2 (0.651)	0.0335	0.996
GO:0031086~nuclear-transcribed mRNA catabolic process, deadenylation-independent decay	2 (0.651)	0.0335	0.996
GO:1902172~regulation of keratinocyte apoptotic process	2 (0.651)	0.0335	0.996
GO:0002548~monocyte chemotaxis	4 (1.30)	0.0338	0.996
GO:0050729~positive regulation of inflammatory response	5 (1.63)	0.0351	0.996
GO:0045444~fat cell differentiation	5 (1.63)	0.0351	0.996
GO:0030334~regulation of cell migration	5 (1.63)	0.0367	0.996
GO:0007596~blood coagulation	8 (2.61)	0.0370	0.996
GO:0000122~negative regulation of transcription from RNA polymerase II promoter	20 (6.51)	0.0373	0.996
GO:0032870~cellular response to hormone stimulus	4 (1.30)	0.0403	0.996
GO:0045638~negative regulation of myeloid cell differentiation	3 (0.977)	0.0403	0.996
GO:0035556~intracellular signal transduction	13 (4.23)	0.0421	0.996

GO:0043153~entrainment of circadian clock by photoperiod	3 (0.977)	0.0443	0.996
GO:0030168~platelet activation	6 (1.95)	0.0457	0.996
GO:0006886~intracellular protein transport	9 (2.93)	0.0479	0.996
GO:0042346~positive regulation of NF-kappaB import into nucleus	3 (0.977)	0.0485	0.996
GO:0007254~JNK cascade	4 (1.30)	0.0499	0.996
GO:0006629~lipid metabolic process	7 (2.28)	0.0508	0.996
GO:0034113~heterotypic cell-cell adhesion	3 (0.977)	0.0527	0.996
GO:0001954~positive regulation of cell-matrix adhesion	3 (0.977)	0.0527	0.996
GO:0006935~chemotaxis	6 (1.95)	0.0563	0.996
GO:0043547~positive regulation of GTPase activity	16 (5.21)	0.0577	0.996
GO:0007422~peripheral nervous system development	3 (0.977)	0.0616	0.996
GO:0019915~lipid storage	3 (0.977)	0.0616	0.996
GO:0048146~positive regulation of fibroblast proliferation	4 (1.30)	0.0632	0.996
GO:0050859~negative regulation of B cell receptor signaling pathway	2 (0.651)	0.0660	0.996
GO:0016032~viral process	10 (3.26)	0.0686	0.996
GO:0001570~vasculogenesis	4 (1.30)	0.0690	0.996
GO:0019432~triglyceride biosynthetic process	3 (0.977)	0.0710	0.996
GO:0051928~positive regulation of calcium ion transport	3 (0.977)	0.0710	0.996
GO:0046777~protein autophosphorylation	7 (2.28)	0.0724	0.996
GO:0006661~phosphatidylinositol biosynthetic process	4 (1.30)	0.0749	0.996

GO:0070373~negative regulation of ERK1 and ERK2 cascade	4 (1.30)	0.0749	0.996
GO:0090383~phagosome acidification	3 (0.977)	0.0759	0.996
GO:0009612~response to mechanical stimulus	4 (1.30)	0.0780	0.996
GO:0032715~negative regulation of interleukin-6 production	3 (0.977)	0.0808	0.996
GO:0048247~lymphocyte chemotaxis	3 (0.977)	0.0808	0.996
GO:0051881~regulation of mitochondrial membrane potential	3 (0.977)	0.0808	0.996
GO:0070431~nucleotide-binding oligomerization domain containing 2 signaling pathway	2 (0.651)	0.0818	0.996
GO:0015833~peptide transport	2 (0.651)	0.0818	0.996
GO:0010656~negative regulation of muscle cell apoptotic process	2 (0.651)	0.0818	0.996
GO:0042634~regulation of hair cycle	2 (0.651)	0.0818	0.996
GO:1990441~negative regulation of transcription from RNA polymerase II promoter in response to endoplasmic reticulum stress	2 (0.651)	0.0818	0.996
GO:2001046~positive regulation of integrin-mediated signaling pathway	2 (0.651)	0.0818	0.996
GO:0001933~negative regulation of protein phosphorylation	4 (1.30)	0.0843	0.996
GO:0007229~integrin-mediated signaling pathway	5 (1.63)	0.0871	0.996
GO:0006897~endocytosis	6 (1.95)	0.0873	0.996
GO:0010468~regulation of gene expression	5 (1.63)	0.0896	0.996
GO:2000379~positive regulation of reactive oxygen species	3 (0.977)	0.0910	0.996

	metabolic process			
	GO:0006611~protein export from nucleus	3 (0.977)	0.0910	0.996
	GO:0044344~cellular response to fibroblast growth factor stimulus	3 (0.977)	0.0910	0.996
	GO:0007050~cell cycle arrest	6 (1.95)	0.0914	0.996
	GO:0030335~positive regulation of cell migration	7 (2.28)	0.0921	0.996
	GO:0007166~cell surface receptor signaling pathway	9 (2.93)	0.0945	0.996
	GO:0030100~regulation of endocytosis	3 (0.977)	0.0962	0.996
	GO:0045577~regulation of B cell differentiation	2 (0.651)	0.0973	0.996
	GO:0045084~positive regulation of interleukin-12 biosynthetic process	2 (0.651)	0.0973	0.996
	GO:0046578~regulation of Ras protein signal transduction	2 (0.651)	0.0973	0.996
	GO:0070245~positive regulation of thymocyte apoptotic process	2 (0.651)	0.0973	0.996
	GO:0032680~regulation of tumor necrosis factor production	2 (0.651)	0.0973	0.996
	GO:0001701~in utero embryonic development	7 (2.28)	0.0983	0.996
UP	GO:0006614~SRP-dependent cotranslational protein targeting to membrane	37 (24.5)	1.12E-52	7.11E-50
	GO:0000184~nuclear-transcribed mRNA catabolic process, nonsense-mediated decay	38 (25.2)	3.89E-50	1.23E-47
	GO:0019083~viral transcription	37 (24.5)	2.29E-49	3.88E-47
	GO:0006413~translational initiation	39 (25.8)	2.45E-49	3.88E-47
	GO:0006364~rRNA processing	38 (25.2)	1.18E-39	1.49E-37

GO:0006412~translation	37 (24.5)	2.28E-35	2.40E-33
GO:0031424~keratinization	16 (10.6)	1.16E-20	1.05E-18
GO:0008544~epidermis development	18 (11.9)	1.31E-19	1.04E-17
GO:0030216~keratinocyte differentiation	16 (10.6)	2.48E-17	1.75E-15
GO:0018149~peptide cross-linking	12 (7.95)	1.45E-13	9.21E-12
GO:0002181~cytoplasmic translation	8 (5.30)	7.12E-10	4.10E-08
GO:0000028~ribosomal small subunit assembly	5 (3.31)	1.33E-05	0.000702
GO:0042274~ribosomal small subunit biogenesis	4 (2.65)	0.000252	0.0123
GO:0061436~establishment of skin barrier	4 (2.65)	0.000363	0.0165
GO:1902600~hydrogen ion transmembrane transport	5 (3.31)	0.00138	0.0567
GO:0098609~cell-cell adhesion	9 (5.96)	0.00143	0.0567
GO:0042254~ribosome biogenesis	4 (2.65)	0.00287	0.107
GO:0002009~morphogenesis of an epithelium	3 (1.99)	0.00532	0.188
GO:0045104~intermediate filament cytoskeleton organization	3 (1.99)	0.00611	0.194
GO:0042633~hair cycle	3 (1.99)	0.00611	0.194
GO:0006120~mitochondrial electron transport, NADH to ubiquinone	4 (2.65)	0.00686	0.207
GO:0006091~generation of precursor metabolites and energy	4 (2.65)	0.00853	0.242
GO:0006414~translational elongation	3 (1.99)	0.00877	0.242
GO:0042776~mitochondrial ATP synthesis coupled proton transport	3 (1.99)	0.0118	0.300
GO:0000027~ribosomal large subunit assembly	3 (1.99)	0.0118	0.300

GO:0032981~mitochondrial respiratory chain complex I assembly	4 (2.65)	0.0137	0.333
GO:0071624~positive regulation of granulocyte chemotaxis	2 (1.32)	0.0158	0.371
GO:0042273~ribosomal large subunit biogenesis	3 (1.99)	0.0166	0.375
GO:0006336~DNA replication-independent nucleosome assembly	3 (1.99)	0.0179	0.391
GO:0031069~hair follicle morphogenesis	3 (1.99)	0.0192	0.406
GO:0006754~ATP biosynthetic process	3 (1.99)	0.0220	0.45
GO:0045110~intermediate filament bundle assembly	2 (1.32)	0.0236	0.467
GO:0006335~DNA replication-dependent nucleosome assembly	3 (1.99)	0.0264	0.508
GO:0051343~positive regulation of cyclic-nucleotide phosphodiesterase activity	2 (1.32)	0.0390	0.727
GO:0006986~response to unfolded protein	3 (1.99)	0.0436	0.775
GO:0006469~negative regulation of protein kinase activity	4 (2.65)	0.0440	0.775
GO:0034080~CENP-A containing nucleosome assembly	3 (1.99)	0.0455	0.780
GO:0001649~osteoblast differentiation	4 (2.65)	0.0497	0.828
GO:0021762~substantia nigra development	3 (1.99)	0.0575	0.935
GO:0045814~negative regulation of gene expression, epigenetic	3 (1.99)	0.0596	0.945
GO:0000463~maturation of LSU-rRNA from tricistronic rRNA transcript (SSU-rRNA, 5.8S rRNA, LSU-rRNA)	2 (1.32)	0.0691	0.981

GO:0070301~cellular response to hydrogen peroxide	3 (1.99)	0.0750	0.981
GO:0017148~negative regulation of translation	3 (1.99)	0.0772	0.981
GO:0045815~positive regulation of gene expression, epigenetic	3 (1.99)	0.0866	0.981
GO:0031581~hemidesmosome assembly	2 (1.32)	0.0910	0.981
GO:0000086~G2/M transition of mitotic cell cycle	4 (2.65)	0.0951	0.981

GO terms were yielded by DAVID (Category: GOTERM_BP_DIRECT) in each cohort using downregulated or upregulated DEGs.

Supplementary Table S5. GO terms of biological process obtained using common DEGs in both cohorts.

Term	Count (%)	*p*-value	FDR
GO:0006954~inflammatory response	4 (23.5)	0.00424	0.582
GO:0071621~granulocyte chemotaxis	2 (11.8)	0.00446	0.582
GO:0002548~monocyte chemotaxis	2 (11.8)	0.0369	1.00
GO:0031532~actin cytoskeleton reorganization	2 (11.8)	0.0412	1.00
GO:0019722~calcium-mediated signaling	2 (11.8)	0.0446	1.00
GO:0070098~chemokine-mediated signaling pathway	2 (11.8)	0.0616	1.00
GO:0030334~regulation of cell migration	2 (11.8)	0.0641	1.00
GO:0030216~keratinocyte differentiation	2 (11.8)	0.0658	1.00
GO:0008544~epidermis development	2 (11.8)	0.0733	1.00

GO terms were yielded by DAVID (Category: GOTERM_BP_DIRECT) using the common DEGs in both cohorts.

Supplementary Table S6. Correlation between SSL-RNA expression and levodopa dose (top10 genes).

Positively correlated genes				Negatively correlated genes			
Symbol	rho	*p*-value	FDR	Symbol	rho	*p*-value	FDR
FADS2	0.566	0.0000415	0.194	*ENO1*	−0.502	0.000373	0.558
UBIAD1	0.512	0.000277	0.558	*CD52*	−0.472	0.000917	0.558
PLIN5	0.464	0.00115	0.558	*SUB1*	−0.464	0.00118	0.558
MARCH5	0.451	0.00166	0.558	*RGS1*	−0.462	0.00122	0.558
HILPDA	0.449	0.00174	0.558	*LDHA*	−0.461	0.00125	0.558
RUSC2	0.448	0.00181	0.558	*PDCD5*	−0.457	0.00143	0.558
H1F0	0.442	0.00213	0.558	*ACSL5*	−0.438	0.00232	0.558
LOC100093631	0.429	0.00290	0.558	*HSP90B1*	−0.437	0.00238	0.558
PNPLA5	0.429	0.00292	0.558	*POLR2E*	−0.430	0.00282	0.558
FYCO1	0.424	0.00329	0.558	*PFDN5*	−0.428	0.00301	0.558

Correlation between SSL-RNA expression level [log2 (normalized count + 1)] and levodopa dose (mg) was calculated using Spearman's correlation coefficient (rho).
